# Effects of Thawing Methods on the Roasting Quality and Flavor Profiles of Reduced-Salt Marinated Large Yellow Croaker (*Larimichthys crocea*)

**DOI:** 10.3390/foods14244213

**Published:** 2025-12-08

**Authors:** Yijia Deng, Shumin Liu, Shengjun Chen, Yaqi Kou, Xin Liang, Xinyi Jiang, Chen Wang, Ravi Gooneratne, Jianrong Li

**Affiliations:** 1 College of Food Science and Engineering, Lingnan Normal University, Zhanjiang 524048, China; ikea7713@163.com (Y.D.); 13763094052@163.com (Y.K.); 19128026096@163.com (X.L.); xinyier0101@163.com (X.J.); 18642326533@163.com (C.W.); 2 National & Local Joint Engineering Research Center of Storage, Processing and Safety Control Technology for Fresh Agricultural and Aquatic Products (Sub-Center), Zhanjiang 524048, China; 3 Key Laboratory of Aquatic Product Processing, Ministry of Agriculture and Rural Affairs, South China Sea Fisheries Research Institute, Chinese Academy of Fishery Sciences, Guangzhou 510300, China; chenshengjun@scsfri.ac.cn; 4 Department of Wine, Food and Molecular Biosciences, Faculty of Agriculture and Life Sciences, Lincoln University, Lincoln 7647, New Zealand; ravi.gooneratne2@lincolnuni.ac.nz; 5 College of Food Science and Engineering, Bohai University, Jinzhou 121013, China

**Keywords:** large yellow croaker, reduced-salt marinated, thawing methods, sensory properties, electronic nose-GC-IMS

## Abstract

This study investigated the impact of thawing methods on the roasting quality and flavor of reduced-salt marinated large yellow croaker to optimize processing protocols for frozen products. Three thawing methods, low-temperature thawing (LTT), room-temperature thawing (RTT), and flowing-water thawing (FWT), were systematically evaluated. Freshly marinated (FM) and non-thawed (WT) samples served as controls. Key parameters, including thawing efficiency, physicochemical properties, texture, color, sensory attributes, and volatile organic compounds (VOCs), were analyzed. The results showed that FWT achieved the fastest thawing (14.67 min), significantly outperforming RTT (32.57 min) and LTT (591 min) (*p* < 0.05). Moisture content and springiness remained stable across treatments (*p* > 0.05). For color parameters, lightness (L*), yellowness (b*), and browning index (BI) showed no significant variations (*p* > 0.05), while the total color difference (ΔE) was significantly affected by thawing methods (*p* < 0.05). FWT exhibited the lowest salt retention (3.49 g/100 g), a 18.8% reduction compared to WT (4.30 g/100 g). Texture analysis revealed that FWT samples maintained optimal hardness and chewiness, with sensory scores second only to WT. Volatile profiling identified distinct “thermal–oxygen–temporal” effects, referring to the respective influences of heating conditions, oxidative environments, and processing time on flavor compound formation. RTT and WT treatments significantly increased the relative 1-propanethiol and 5-methyl-2-furanmethanol (>10% increase) contents, respectively, and markedly reduced the 2-butanol levels (<0.3%) due to volatilization losses. GC-IMS and electronic nose analysis established a robust correlation network among three major VOC clusters (aldehydes/alcohols, esters/acid/sulfides, and ketones), with sensory scores showing strong positive correlations with the alkane- and aromatic-sensitive sensors (W5C/W1C) of the electronic nose (r > 0.90) and negative correlations with other sensors (r < −0.70). These findings demonstrate that FWT offers the best balance of efficiency, salt reduction, and sensory quality, making it a superior method for reduced-salt marinated large yellow croaker industrial applications.

## 1. Introduction

The large yellow croaker (*Larimichthys crocea*), an economically important species of the Sciaenidae family, represents one of the most iconic marine aquaculture products in China and East Asia [1,2]. Renowned for its golden-yellow skin, delicate texture, low cholesterol content, and abundant polyunsaturated fatty acids, this species holds significant commercial and nutritional value [3]. However, its production chain faces substantial challenges, including stress-induced mortality during cultivation and transportation, lipid oxidation during storage, and microbial spoilage, with all these limiting shelf-life [4,5]. Current commercial preservation methods include ice storage, freezing, and salting, with salt-curing being most common due to its simplicity and effectiveness in extending shelf-life [6,7]. Salting not only minimizes drip loss during freezing but also enhances palatability through flavor compound transformation [6,7].

The growing emphasis on healthy diets has raised concerns about traditional high-salt curing processes [8]. The World Health Organization’s (WHO’s) daily sodium intake recommendation is <2 g (equivalent to 5 g salt) [9]. However, conventional salted seafood products are so high in salt that even a single serving can contribute a substantial portion to this daily limit. For instance, the Chinese industry standard for salted large yellow croaker (SC/T 3216-2016) permits a maximum salt content of up to 6 g/100 g, and traditional artisanal processes can even exceed 8–10 g/100 g [10,11]. This drives the research focus toward reduced-salt alternatives that maintain both sensory quality and frozen stability while reducing the sodium content. In this context, the term ‘reduced-salt’ used in this study signifies that our pickling process achieves a final salt content of 3.5–4.3 g/100 g, representing a 28–42% reduction from standard levels. More importantly, our pickling process utilizes a NaCl-KCl mixture (7:3 mass ratio), replacing 30% of the sodium to achieve a much greater reduction in sodium content (50–59% reduction from standard levels) alongside the total salt reduction, thereby directly addressing health concerns associated with high sodium intake.

The quality of freezing preservation largely depends on subsequent thawing processes. Improper thawing can lead to drip loss, microbial proliferation, and accelerated lipid/protein oxidation through synergistic enzymatic and microbial action, ultimately causing structural deterioration and flavor degradation [11,12,13]. The effectiveness of various thawing methods, including low-temperature (LTT), room-temperature (RTT), flowing-water (FWT), ultrasound, microwave, infrared, and radio frequency thawing, varies in different fish species [14,15]. Cold-air thawing optimally preserves tilapia fillet quality [16], brine–ultrasound thawing suits cuttlefish [12], LTT maintains rainbow trout sashimi quality, and FWT improves water-holding capacity [17] and preserves mackerel muscle integrity [18], indicating species-specific thawing strategies.

Many studies have focused on the optimization of thawing of un-salted frozen products, while there are only a few reports on the thawing characteristics of reduced-salt marinated frozen aquatic products, and most of these lack systematic evaluation of the quality of thawed and subsequently processed products. To address these gaps, three thawing methods suitable for home and catering scenarios (LTT, RTT, FWT) were selected to systematically investigate their effects on multiple key indicators of reduced-salt marinated yellow croaker roasted products, including moisture migration patterns, salt distribution characteristics, texture properties, color stability, and flavor component composition. Fresh marinated and immediately roasted (FM) and frozen direct roasted (WT) samples acted as controls. This research will provide a scientific basis for the development of pre-prepared croaker dishes and a significant guide for standardized operations to the food industry.

## 2. Materials and Methods

### 2.1. Materials and Reagents

Fresh large yellow croaker (*Larimichthys crocea*; Naozhou strain) were harvested from Naozhou Island coastal waters in Zhanjiang, Guangdong Province, China (20.69935° N, 110.76061° E) and immediately transported on ice to the laboratory. All chemicals used were of analytical grade; refined non-iodized salt (Hunan Snowsky Salt Technology Development Co., Ltd., Changsha, China), zinc acetate, acetic acid, sodium tetraborate, potassium ferrocyanide, potassium chromate, and silver nitrate (Shandong Keyuan Biochemical Co., Ltd., Jinan, China). Potassium chloride (Lianyungang Kede Food Ingredients Co., Ltd., Nanjing, China) was food-grade. The fish were processed within 6 h post-capture to ensure optimal freshness for subsequent experiments.

### 2.2. Sample Preparation

The reduced-salt pickling solution was prepared by mixing sodium chloride and potassium chloride at a 7:3 mass ratio in boiled tap water cooled to room temperature, achieving a final chloride concentration of 12% (*w*/*v*). Freshly caught yellow croaker were processed using standardized protocols: (1) scaling and evisceration, (2) longitudinal splitting along the dorsal ridge to obtain two symmetrical fillets (1.5 cm thickness), (3) thorough rinsing with running water to remove surface mucus and impurities, and (4) surface drying with kitchen paper. Prepared fillets were weighed and vacuum-sealed with pickling solution in polyethylene composite bags at a 1:1.2 (*m*/*v*) fish-to-solution ratio. The sealed samples were marinated at 4 °C for 36 h. After pickling, the bags were opened, and the fish fillets were removed and surface-dried with kitchen paper. The dried fillets were then vacuum-packaged and stored at −20 °C for ≥7 days to ensure complete freezing before subsequent experiments.

### 2.3. Thawing and Thermal Processing (Roasting)

Frozen reduced-salt marinated large yellow croaker samples (−20 °C) in their vacuum packaging were subjected to three standardized thawing protocols: (1) room-temperature thawing (RTT) at 25 °C ambient conditions, (2) low-temperature thawing (LTT) in a 4 °C refrigerated environment, and (3) flowing-water thawing (FWT) using continuously circulating tap water (18 °C) at a controlled flow rate of 50 mL/s in stainless steel containers. The thawing process was uniformly monitored using a calibrated probe thermometer (BD-TC-SC, Shenzhen, China) with an accuracy of ±0.5 °C. The probe was inserted through the vacuum packaging into the geometric center of each fillet, with thawing termination based on reaching a 4 °C core temperature [19]. Following thawing, all samples were subjected to standardized thermal processing in an electric oven at 180 °C for 25 min to ensure consistent cooking conditions across experimental groups. This protocol maintained critical experimental controls, including precise temperature regulation (±1 °C), flow rate calibration (±5%), and uniform sample geometry (1.5 cm thickness), to minimize variability.

### 2.4. Analytical Methods for Moisture and Salt Content Determination

The moisture content was determined using the standard oven-drying method [20]. Precisely weighed samples (~2 g) were dried in a forced-air oven at 105 ± 2 °C to constant weight (24–48 h). The moisture content was calculated as the percentage weight loss relative to the initial sample mass. Salt chloride concentration was quantitatively analyzed using the Volhard titration method [21], which involved (i) sample homogenization in deionized water, (ii) precipitation of chloride ions with excess silver nitrate under acidic conditions, and (iii) back-titration of residual silver ions with potassium thiocyanate using ferric ammonium sulfate as the indicator. All measurements were performed in triplicate, with results expressed as grams of chloride per 100 grams of sample (g/100 g, wet basis).

### 2.5. Texture Profile Analysis (TPA)

Texture analysis was performed according to the method described by Zhang et al. [22] with modifications. Following thermal processing, fish muscle samples were precisely cut into uniform cubes (2 × 2 × 1 cm) and analyzed using a texture analyzer (Food Technology Corporation, Sterling, VA, USA) in texture profile analysis (TPA) mode. The following parameters were measured: hardness (peak force during first compression), adhesiveness (negative force area), cohesiveness (ratio of second to first compression areas), springiness (height recovery after compression), gumminess (hardness × cohesiveness), and chewiness (gumminess × springiness). A P/75 cylindrical probe (Food Technology Corporation, Sterling, VA, USA) at 1.0 mm/s with 30% compression (0.05 N trigger force) was used, collecting data at 200 Hz. Six replicates per group were tested within 10 min of 25 °C equilibration, following instrument calibration with a 5 kg load cell. Parameters were optimized through preliminary trials to ensure measurable deformation without structural damage.

### 2.6. Color Measurement

Color analysis was performed using a calibrated chroma meter (SC-80, Beijing, China) following standardized protocols [23]. Prior to measurement, the instrument was calibrated with black and white reference tiles to ensure accuracy. Following thermal processing, uniform fish samples (1 × 1 × 1 cm) were prepared and analyzed in triplicate to determine three key color parameters: L* (lightness/darkness), a* (redness/greenness), and b* (yellowness/blueness). Total color difference (ΔE) and browning index (BI) were calculated by equations as follows:

(1) $$\Delta E=\sqrt{{\left(\Delta L^{*}\right)}^{2}+{\left(\Delta a^{*}\right)}^{2}+{\left(\Delta b^{*}\right)}^{2}}$$

(2) $$\mathrm{BI}=\frac{100\left(\frac{a^{*}+1.75L^{*}}{5.645L^{*}+a^{*}-3.012b^{*}}-0.31\right)}{0.172}$$

where ΔL*, Δa*, and Δb* represent the differences in L*, a*, and b* values, respectively, between the sample and the fresh marinated and immediately roasted (FM) control.

Results from the three replicate measurements were averaged to obtain representative values for each sample group.

### 2.7. Sensory Evaluation

A trained sensory panel consisting of 10 members (5 male, 5 female) aged 20–30 years from the Food Processing and Safety research team conducted the evaluation using a modified version of Armenteros et al.’s method [24]. For sensory evaluation, the roasted fish samples were prepared following the same thermal processing protocol described in Section 2.3. Immediately after roasting and brief cooling, the fish was portioned into standardized, rectangular pieces (approximately 3 cm × 4 cm) with skin retained but all bones removed. Each panelist received a single, randomly coded sample cube served at approximately 40 °C in a white plastic dish.

Prior to assessment, all samples were randomized and re-coded to eliminate bias. Panelists evaluated four key attributes of the reduced-salt marinated large yellow croaker: appearance, aroma/flavor, texture, and mouthfeel, using the standardized scoring criteria detailed in Table S1 (Supplementary Materials). Each attribute was scored on a 25-point scale. The comprehensive score was calculated as the sum of the scores from all four attributes, resulting in a maximum possible score of 100 points. Each panelist received training on the evaluation protocol and attribute definitions before formal testing to ensure consistent interpretation of the scoring scale. Evaluations were conducted in individual sensory booths under controlled environmental conditions (temperature: 22 ± 1 °C, relative humidity: 50 ± 5%, white lighting).

### 2.8. Electronic Nose Analysis

Volatile compound profiling was conducted using a PEN3 electronic nose system (AirSence Corp., Mecklenburg, Germany) based on the protocol of Sheng et al. [25]. The system was equipped with an array of 10 metal oxide sensors (detailed specifications in Table S2). Following thermal processing, the homogenized cooked fish samples was homogenized. Then, 5 g of the homogenate was sealed in 20 mL headspace vials. Samples underwent thermal equilibration at 60 °C for 30 min in a water bath, followed by 30 min stabilization at ambient temperature (25 ± 1 °C) before analysis. Operational parameters included the following: 100 s sampling time, 100 s purge time, and data acquisition at 1 Hz frequency. Principal component analysis (PCA) was performed using the built-in proprietary Winmuster software (version 1.6.2) of the PEN3 electronic nose system with stabilized signals from 90–92 s, and radar plots were generated using 91 s response data. Between samples, the system was purged with filtered air to maintain baseline stability (<5% signal variation).

### 2.9. Gas Chromatography–Ion Mobility Spectrometer (GC-IMS) Analysis

Volatile organic compounds (VOCs) were characterized using a GC-IMS (flavorSpec^®^, Gesellschaft für Analytische Sensorsysteme GmbH, Dortmund, Germany) using an established protocol [26]. For analysis, 2 g of homogenized fish sample was sealed in 20 mL headspace vials and incubated at 60 °C for 15 min with constant agitation (500 rpm). Automated headspace injection (500 μL) was performed at 65 °C into a GC system equipped with an MXT-5 column (15 m × 0.53 mm) maintained at 60 °C, using high-purity nitrogen as the carrier gas at 5 mL/min. The IMS unit operated with a 45 °C drift tube temperature and nitrogen drift gas, applying a 400 V/cm electric field across a 5 cm drift tube at a 150 mL/min flow rate. Each sample was analyzed in triplicate, with data acquired and processed using the manufacturer’s software (v2.2.1). System performance was validated using 1-butanol standards. Blank runs were conducted between samples to ensure measurement integrity. This configuration provided high-sensitivity detection of volatile flavor compounds while maintaining excellent reproducibility (RSD < 5% for retention time and drift time).

### 2.10. Statistical Analysis

All experiments were conducted with a minimum of three independent replicates (n ≥ 3), with data expressed as mean ± standard deviation. Graphical representations were generated using Origin 2024 (OriginLab Corporation, Northampton, MA, USA). Statistical significance was assessed through one-way analysis of variance (ANOVA) followed by Fisher’s Least Significant Difference (LSD) post hoc test (SPSS v27.0, IBM Corp., Armonk, NY, USA), with *p*-values < 0.05 considered statistically significant. For GC-IMS data analysis, the VOCal software (v0.4.03, G.A.S. GmbH, Dortmund, Germany) was employed to process volatile compound profiles, including peak alignment, normalization, and multivariate statistical evaluation. Prior to ANOVA, data normality (Shapiro–Wilk’s test) and homogeneity of variance (Levene’s test) were verified to ensure parametric test assumptions were met.

## 3. Results and Discussion

### 3.1. Effect of Thawing Methods on Thawing Time, Moisture Content, and Salt Concentration

The thawing process plays a critical role in determining the final quality of frozen aquatic products, as it directly influences heat–mass transfer dynamics and subsequent physicochemical changes [27]. Significant differences (*p* < 0.05) were observed in thawing time among the three conventional thawing methods (Table 1), with FWT exhibiting the shortest duration (14.67 ± 1.53 min), followed by RTT, (32.57 ± 3.08 min) and LTT (591 ± 18.33 min). This hierarchical pattern can be attributed to fundamental differences in heat transfer mechanisms: While both LTT and RTT rely on natural convective heat transfer through air medium, the higher thermal conductivity of air at 25 °C (RTT) compared to 4 °C (LTT) accounts for their respective time differentials [28,29]. In contrast, FWT achieved superior efficiency through forced convective heat transfer, where continuous water flow (20 °C, 50 mL/s) maintained an optimal temperature gradient at the product surface, thereby accelerating ice crystal dissolution [30].

Notably, despite these temporal disparities, all thawed samples maintained comparable moisture content (66.45–68.61 g/100 g) in both the fresh marinated (FM, 65.87 g/100 g) and without-thawing (WT, 66.73 g/100 g) controls (*p* > 0.05) (Table 1). This result aligns with previous findings by Jiang et al. [31,32], who reported that reduced-salt marination enhances water-holding capacity by modifying muscle microstructure and ice crystal morphology. The structural stabilization effect of the NaCl-KCl brine may explain the remarkable moisture retention across all thawing treatments, since the formed protein matrix effectively restricts water migration during phase transition.

However, the salt concentration exhibited significant method-dependent variation (*p* < 0.05), with FWT demonstrating the lowest value (3.49 ± 0.01 g/100 g), a 18.8% reduction compared to WT (4.30 ± 0.02 g/100 g) (Table 1). This can be mechanistically explained by the following: (1) osmotic equilibration during FWT, where the external low-salinity environment (flowing water) creates a concentration gradient that drives intracellular salt diffusion outward; (2) minimal salt retention in WT due to protein-denaturation-induced gel network formation during direct roasting, which physically traps salts within the muscle matrix; and (3) intermediate salt levels in FM (4.21 ± 0.04 g/100 g) reflecting baseline marination effects without freeze–thaw-induced solute redistribution [33]. These findings collectively highlight that thawing methods negligibly affect water retention but substantially modulate salt distribution patterns, a crucial consideration for developing reduced-sodium seafood products.

### 3.2. Effect of Thawing Methods on Texture Properties

Texture profile analysis revealed significant variations in the mechanical properties of reduced-salt marinated large yellow croaker under different thawing treatments (Table 2). The textural parameters, including hardness, adhesiveness, cohesiveness, gumminess, and chewiness, showed significant differences between treatments (*p* < 0.05), while springiness was unaffected (*p* > 0.05). While RTT and WT showed reduced chewiness compared to FM, FWT maintained a chewiness value that was comparable to or higher than these other thawing methods, indicating better texture preservation. This textural modification pattern can be attributed to the cryo-mechanical effects during frozen storage, where ice crystal formation induces myofibril fragmentation and intercellular space expansion. Such alterations lead to passive toughening of muscle tissue, compromising its structural integrity, as evidenced by decreased cohesiveness and chewiness [33,34,35]. Furthermore, frozen storage likely promotes conformational changes in myofibrillar proteins and exudation of sarcoplasmic proteins, enhancing surface adhesiveness and gumminess [34,35]. In contrast, LTT-treated samples exhibited inferior textural properties across all measured parameters except springiness. The prolonged thawing duration (591 ± 18.33 min) at 4 °C may facilitate ice recrystallization, causing additional mechanical damage to muscle. These processes could lead to the formation of heterogeneous protein aggregates under cryo-mechanical stress, ultimately accelerating protein denaturation and textural deterioration [36,37,38].

Interestingly, all treatment groups showed comparable springiness (1.80–2.15 mm), suggesting that this parameter was predominantly influenced by the subsequent roasting process rather than the thawing method. The uniform thermal denaturation of actomyosin complex and inactivation of ATPase during roasting at 180 °C likely contributed to the muscle fiber contraction across all samples [33,39]. These findings demonstrate that while the thawing method significantly impacts multiple textural attributes, springiness is primarily determined by the roasting process.

### 3.3. Effect of Thawing Methods on Color Characteristics

Color stability represents a critical quality parameter that significantly influences consumer acceptance of frozen–thawed fish products [40]. The thawing methods exhibited differential effects on various color parameters of reduced-salt marinated large yellow croaker (Table 3). While lightness (L*) and yellowness (b*) showed no significant variations among treatments (*p* > 0.05), notable differences were observed in redness (a*) and the total color difference (ΔE) (*p* < 0.05). In contrast, the browning index (BI) remained consistent across all treatments (*p* > 0.05). The maintenance of consistent L* and b* values across all treatments suggests that the freeze–thaw processes did not induce substantial myofibrillar collapse or moisture loss that significantly impacted light scattering properties, a finding that is corroborated by the stable moisture content (65.87–68.61 g/100 g) [41,42]. However, the significant differences in ΔE indicate that the combined variations in L*, a*, and b*—primarily driven by the pronounced increase in a* (redness)—led to perceptible overall color deviations from the fresh marinated and immediately roasted (FM) control. Notably, the FWT and WT samples exhibited the largest ΔE values (4.20 ± 0.27 and 3.54 ± 0.68, respectively), indicating that their color profiles differed most from the FM control. Despite these ΔE variations, the non-significant BI values indicate that the thermal-induced browning extent remained consistent regardless of thawing method. This color stability may be attributed to the protective effect of reduced-salt marination, which helps to maintain the structural integrity during phase transitions.

Interestingly, all frozen–thawed samples displayed significantly higher a* values (3.32–5.05) compared to FM controls (3.24 ± 0.04), with the FWT-treated samples exhibiting a pronounced redness (5.05 ± 0.15). This can be mechanistically explained by two complementary processes: (i) Freezing-induced protein denaturation promotes carbonyl compound accumulation during frozen storage, which participates in Maillard reactions with amino acids during roasting, enhancing red pigment formation [43,44]. (ii) Rapid thawing under FWT minimizes ice recrystallization damage, preserving pigment integrity and enabling greater surface pigment expression during roasting, which contributes to the higher a* values observed [33,45]. However, the lack of significant differences in BI suggests that these processes did not lead to substantially different levels of overall browning. The LTT-treated samples showed color parameters (L* = 48.05 ± 0.31, a* = 3.32 ± 0.02, b* = 11.85 ± 0.22) comparable to FM, most likely due to the gentle thawing conditions (4 °C, 591 min) that minimized protein structural damage and delayed denaturation kinetics, thereby better preserving the native color profile [45].

The colorimetric findings demonstrate that thawing methods significantly influence redness (a*) development and the total color difference (ΔE) relative to the FM control, primarily through pathways involving protein oxidation and pigment migration during the freeze–thaw phase. However, the consistent browning index (BI) across treatments indicates that the subsequent roasting process predominately determines the final extent of thermal browning, overshadowing the initial differences induced by thawing. These results provide valuable insights for process optimization to achieve desirable color characteristics in prepared fish products.

### 3.4. Effect of Thawing Methods on Sensory Attributes

**Figure 1:**
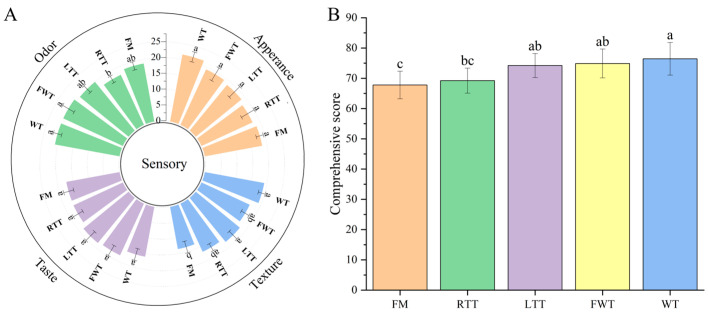
Sensory evaluation radar chart (**A**) and comprehensive score (**B**) of reduced-salt marinated large yellow croaker under different thawing treatments. Different lowercase letters above the bars in (B) indicate significant differences among groups (*p* < 0.05). FM: fresh marinated and immediately roasted, RTT: room-temperature thawing, LTT: low-temperature thawing, FWT: flowing-water thawing, WT: frozen direct roasted.

Sensory evaluation is a critical indicator of product quality and consumer preference for fish products [3]. Thawing methods exerted significant influences on odor, texture, and comprehensive score (calculated as the sum of all four sensory attributes, thus on a 0–100 scale; *p* < 0.05) with no notable effects on appearance and taste (Figure 1). This observation aligns with the colorimetric analysis results, demonstrating consistent appearance across treatments, with taste stability attributed to the pre-fixation of flavor compounds during the marination process [7].

Sensory evaluation revealed distinct patterns between treatments: the WT samples showed the highest comprehensive score (76.4 ± 5.41) but contained an elevated salt content (4.30 ± 0.02 g/100 g) that may pose health risks [8], while LTT and FWT achieved comparable sensory quality (74.2–74.9) with lower salt concentrations (3.49–3.85 g/100 g). Gentle 4 °C thawing during LTT treatment minimized biochemical reactions, while rapid processing (14.67 ± 1.53 min) of the FWT samples may help restrict microbial proliferation, thereby contributing to better preservation of product quality [41]. In contrast, the FM controls demonstrated inferior texture scores due to minimal freezing-induced myofiber contraction, marked softening during heating, and reduced hardness/elasticity (3.60 ± 0.15 N) [33], suggesting that controlled freezing can enhance the structural properties of the final product. These findings demonstrate that proper thawing method selection, particularly FWT, can maintain desirable sensory characteristics while addressing health considerations through reduced salt retention in reduced-salt marinated fish products. Notably, odor scores correlated strongly with specific volatile compounds (e.g., sulfur-containing compounds and aromatic esters) and corresponding electronic nose sensor responses (e.g., W1W/W2W and W5C/W1C). These systematic relationships, which are explored in detail in the subsequent correlation analysis (Section 3.7), demonstrate that thawing methods modulate distinct flavor pathways.

### 3.5. Effect of Thawing Methods on Volatile Organic Compounds (VOCs)

**Figure 2:**
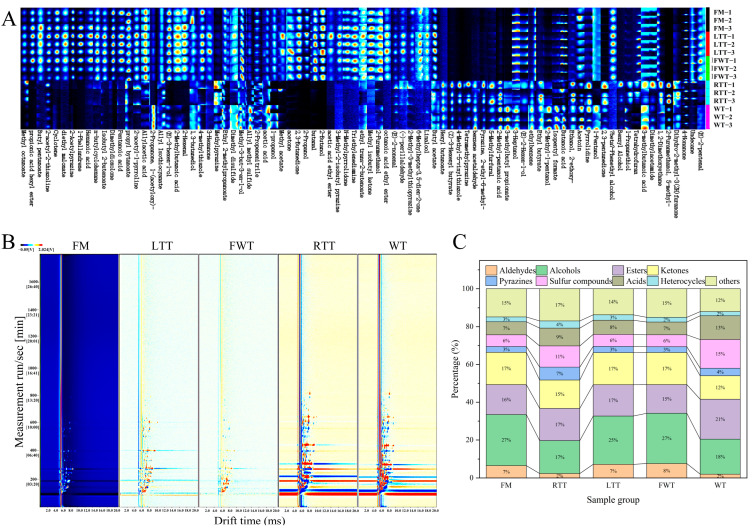
GC-IMS topographic plots and volatile compound distribution in reduced-salt marinated large yellow croaker: (**A**) gallery plot, (**B**) differential plot, (**C**) compositional analysis. FM: fresh marinated and immediately roasted, RTT: room-temperature thawing, LTT: low-temperature thawing, FWT: flowing-water thawing, WT: frozen direct roasted.

GC-IMS analysis revealed significant differences in VOC profiles between treatments (Figure 2A). The analysis identified 84 distinct VOCs, categorized into nine major chemical classes: 7 aldehydes, 15 alcohols, 18 esters, 12 ketones, 6 pyrazines, 6 sulfur-containing compounds, 8 acids, 3 heterocyclic compounds, and 9 miscellaneous compounds. The complete VOC profile, including compound identities and their relative abundances (%), is presented in Table S3. The gallery plot (Figure 2A) demonstrated three distinct clusters: (1) FM, LTT, and FWT that showed similar fingerprint patterns; (2) RTT and WT formed a separate cluster with partial overlap; and (3) a differential plot (Figure 2B) that clearly visualized these relationships, with LTT/FWT exhibiting higher overall VOC retention than FM (red signals), while RTT/WT showed selective compound depletion (blue signals). Composition analysis (Figure 2C) showed that alcohols, ketones, and esters collectively accounted for 58.71–59.88% of total VOCs in FM/LTT/FWT, whereas RTT showed reduced total volatiles (49.41%) and WT displayed a unique profile dominated by esters, alcohols, and sulfur compounds.

**Figure 3:**
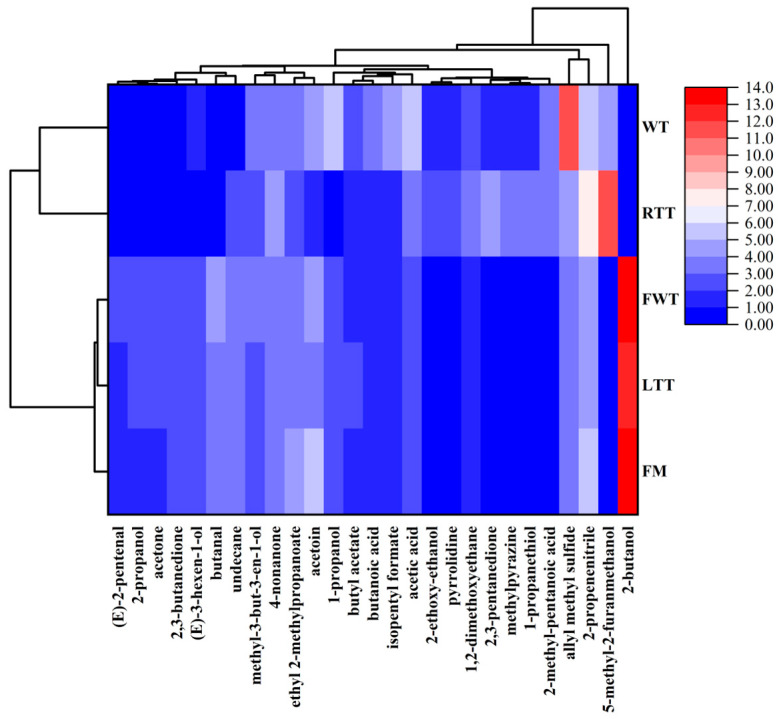
Heatmap analysis of key volatile organic compounds in reduced-salt marinated large yellow croaker across thawing treatments. FM: fresh marinated and immediately roasted, RTT: room-temperature thawing, LTT: low-temperature thawing, FWT: flowing-water thawing, WT: frozen direct roasted.

The cluster heatmap (Figure 3) of 27 major VOCs (relative content > 2%) highlighted key compositional shifts. Most notably, the 2-butanol content decreased dramatically in RTT (0.228%) and WT (0.244%) compared to the other treatments (12.68–13.95%), primarily due to enhanced volatilization under their respective processing conditions. In RTT, prolonged room-temperature exposure (25 °C, 32.57 min) promoted alcohol loss through capillary pumping and convective dissipation [46,47]. WT samples experienced even greater losses through steam stripping during direct high-temperature roasting, where the extreme thermal gradient (>60 °C surface vs. <0 °C core) created ideal conditions for volatile removal [1,47]. Conversely, FM/LTT/FWT effectively preserved 2-butanol through three protective mechanisms: brine immobilization (FM), ice crystal barriers (LTT), and rapid processing with surface cooling (FWT) [1,47,48].

Sulfur compounds exhibited treatment-specific patterns: WT showed elevated allyl methyl sulfide (AMS, 11.02% vs. 3.36–4.27% in others) due to selective retention of this hydrophobic compound (bp 92 °C, log P ≈ 1.8) following removal of more polar volatiles [49,50]. RTT uniquely accumulated 1-propanethiol (3.48% vs. 0.40–0.48% in others) through enzymatic generation during thawing and thermal stabilization in roasting [51,52]. Both RTT and WT also showed marked increases in 5-methyl-2-furanmethanol (5-MFM, 4.44–11.46% vs. 0.30–0.35%), generated through different pathways (oxidative Maillard reactions in RTT versus pyrolytic caramelization in WT) [53]. These findings demonstrate that thawing methods profoundly influence VOC profiles via both physical (volatilization) and chemical (generation) mechanisms, with FWT showing optimal balance between flavor retention and health considerations.

### 3.6. Electronic Nose Analysis of Volatile Flavor Profiles

**Figure 4:**
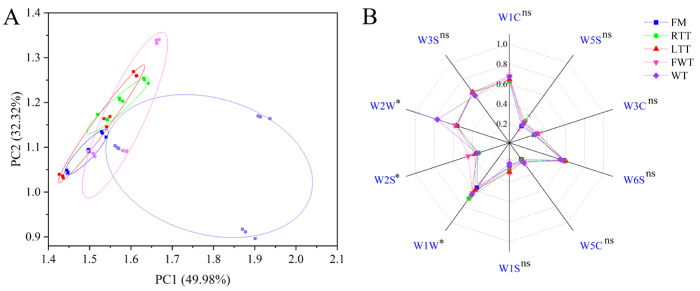
Multivariate analysis of volatile flavor profiles: (**A**) PCA score plot, (**B**) electronic nose sensor response patterns. Sensor designations and their main detection categories are as follows: W1C (aromatic substance), W5S (extremely sensitive to nitrogen oxides), W3C (ammonia, aromatic substance), W6S (hydrogen), W5C (alkanes, aromatic substances), W1S (methane), W1W (sulfur-containing compounds), W2S (alcohols, aldehydes), W2W (aromatic compounds, organic sulfides), and W3S (methane). FM: fresh marinated and immediately roasted, RTT: room-temperature thawing, LTT: low-temperature thawing, FWT: flowing-water thawing, WT: frozen direct roasted. The asterisk (*) represents significant difference (*p* < 0.05); ^ns^ denotes no significant difference.

The electronic nose system, coupled with GC-IMS analysis, provided complementary characterization of volatile compound differences among thawing treatments (Figure 4). Principal component analysis (PCA) of the sensor array data revealed that the first two principal components (PC1 and PC2) collectively explained 81.31% of the total variance (Figure 4A), adequately representing the overall flavor characteristics. The discrimination indices between FM-LTT (0.326), FM-FWT (0.369), and LTT-FWT (0.348) pairs were all below the 0.5 threshold (Table S4), indicating minimal odor differences between these treatments. It is noteworthy that the FWT group exhibited greater variability in the PCA space (Figure 4A), suggesting more heterogeneity in its volatile profile, primarily attributable to inter-fish biological variation rather than sensor inconsistency. Nevertheless, its overall signature remained closer to FM and LTT than to RTT or WT. This finding is consistent with (1) non-significant odor score variations in sensory evaluation (*p* > 0.05) and (2) highly similar VOC fingerprints and compositional profiles in GC-IMS analysis.

The electronic nose sensor response patterns (Figure 4B) provided a deeper, sensor-level explanation for the volatile profile clusters observed in the PCA (Figure 4A). While the overall profiles of FM, LTT, and FWT showed considerable similarity—consistent with the low discrimination indices—statistically significant differences (*p* < 0.05) were pinpointed to three key sensors: W2S, W1W, and W2W. These specific responses directly articulate the subtle differences underlying the group separations. FM samples exhibited a trend for the lowest overall sensor responses, showing significantly lower values in sensors W1W and W2W (*p* < 0.05), indicating minimal aroma, likely due to the absence of freeze–thaw cycles limiting cell membrane disruption, reduced lipid exposure to pro-oxidants (enzymes, iron ions), and lower accumulation of Maillard reaction precursors (amino acids, reducing sugars) [54]. In contrast, FWT demonstrated significantly higher W2S (alcohols/aldehydes) responses (*p* < 0.05), correlating with elevated alcohol (15.8%) and aldehyde (9.2%) levels in GC-IMS, enhanced retention of fresh, fruity aroma compounds, and reduced volatile loss during rapid thawing. RTT and WT showed a pronounced W1W (sulfur compounds) response (*p* < 0.05), compared to other treatments, the generation of characteristic roasted/meaty aromas, and thermal degradation of sulfur-containing amino acids [55]. Additionally, WT uniquely exhibited elevated W2W (aromatics/organic sulfides) responses, reflecting selective enrichment of esters (22.4%) and sulfur compounds (11.0%), high-temperature-induced flavor compounds, and volatile redistribution during direct roasting. These findings demonstrate that while thawing methods induced significant changes in specific aroma pathways—notably alcohols/aldehydes and sulfur compounds—the overall aroma profiles of FM, LTT, and FWT remained closely related. FWT optimally preserved desirable fresh-fruity volatiles while minimizing the formation of roasted/sulfur notes associated with other methods.

The strong concordance between the electronic nose and GC-IMS data (r > 0.89 for key compound classes) validates that thawing methods systematically modify flavor profiles by controlling (1) water migration pathways during phase transitions and (2) thermal reaction intensity during subsequent roasting. These findings demonstrate how processing conditions can be optimized to achieve desired flavor characteristics in reduced-salt marine products.

### 3.7. Multivariate Correlation Analysis of Quality Parameters

**Figure 5:**
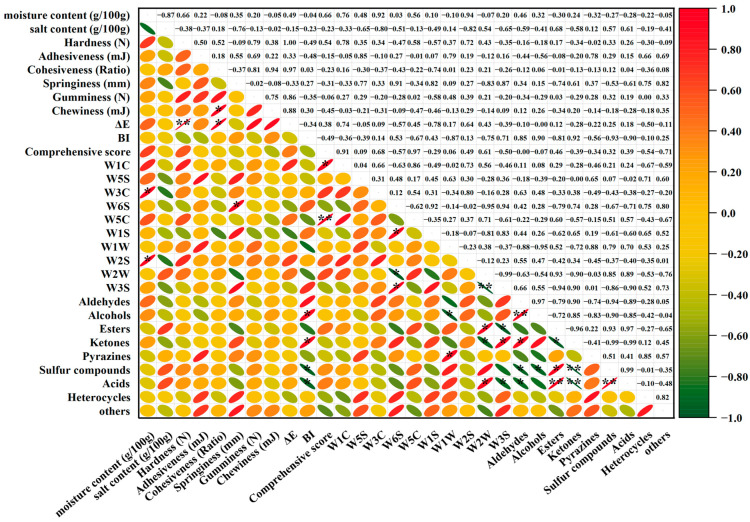
Correlation network between quality parameters of reduced-salt marinated large yellow croaker based on Pearson’ s analysis. ΔE: total color difference, BI: browning index. Sensor designations and their main detection categories are as follows: W1C (aromatic substance), W5S (extremely sensitive to nitrogen oxides), W3C (ammonia, aromatic substance), W6S (hydrogen), W5C (alkanes, aromatic substances), W1S (methane), W1W (sulfur-containing compounds), W2S (alcohols, aldehydes), W2W (aromatic compounds, organic sulfides), and W3S (methane). The asterisk (*) represents significant difference. *: *p* < 0.05; **: *p* < 0.01.

Pearson’s correlation analysis (|r| ≥ 0.8) revealed a complex interaction network among physicochemical, textural, and flavor characteristics (Figure 5). The color–texture axis demonstrated particularly strong associations, with hardness showing near-perfect positive correlation with ΔE (color difference, r ≈ 1.00). Hardness also showed a strong positive correlation with W1C (aromatic sensor, r = 0.78), and ΔE was positively related to W1C (r = 0.74). This triad of relationships establishes a “harder–greater color deviation (ΔE)” paradigm, indicating that textural changes are strongly associated with measured color parameters [56,57]. Simultaneously, moisture content appeared to be a key mediator, exhibiting dual pathways: positive correlations with fresh/roasty aromas (W2S/W3C, r = 0.92–0.94) through water retention mechanisms [58,59] and negative associations with salt content (r = −0.87) that indirectly modulated ester formation (r = 0.68) via salting-out effects [60].

The volatile compound network was organized into three distinct metabolic modules: (1) a fresh–fruity cluster (aldehydes-alcohols, r = 0.97) representing primary oxidation products [61], (2) a roasted–savory complex (esters–acids–sulfur compounds, r > 0.93) characteristic of Maillard/caramelization derivatives in RTT/WT groups [62], and (3) antagonistic ketones (r ≤ −0.96 with other clusters), suggesting competing biochemical pathways [61]. Electronic nose signatures confirmed these groupings, with the W2W response strongly tracking ester–sulfur content (r = 0.85–0.93), while W3S was inversely correlated (r = −0.99), indicating that optimal flavor balance requires simultaneous ester promotion and ketone suppression [63]. These relationships were further reflected in the opposing behavior of W5C/W1C (aromatic) and other sensors, which showed strong positive (r = 0.91–0.97) and negative (r = −0.71) correlations, respectively, with the comprehensive sensory score [64].

The comprehensive sensory score integrated these multidimensional relationships through two primary pathways: (1) a positive quality axis linking moisture, esters, and sulfur compounds to aromatic sensors (W5C/W1C) and (2) a negative axis dominated by undesired volatiles (others). This framework demonstrates how thawing methods can systematically influence quality parameter networks, with FWT optimally positioned by maintaining high moisture (preserving fresh aromas) while limiting salt-driven esterification (avoiding health concerns). The robust correlations (>0.9) between instrumental measures and sensory outcomes validate the use of GC-IMS–electronic nose systems for rapid quality assessment in processed seafood products.

## 4. Conclusions

This study systematically evaluated the effects of different thawing methods on the roasting quality and flavor profiles of reduced-salt marinated large yellow croaker, demonstrating FWT as the optimal processing method achieved by rapid thawing (14.67 min, 90.6–97.5% faster than RTT/LTT), significant salt reduction (3.49 g/100 g, 18.8% lower than WT), and superior quality retention through enhanced salt diffusion due to concentration gradients that maintain moisture content and textural integrity while meeting low-sodium consumer demands. A comprehensive analysis revealed distinct quality modulation mechanisms between treatments: LTT’ s prolonged 4 °C exposure may have contributed to texture deterioration, RTT’ s oxygen-rich environment enriched 1-propanethiol, and WT’ s thermal shock promoted 5-methyl-2-furanmethanol formation, with all frozen treatments showing volatile compound redistribution. Correlation networks established critical quality relationships, including the strong positive correlation between hardness and total color difference (ΔE, |r| > 0.97), moisture–aroma preservation pathways (r > 0.90), and competitive volatile metabolism (r ≤ −0.99), providing a theoretical foundation for future optimization of integrated GC-IMS-E–nose–texture–color evaluation systems to further enhance processing efficiency while balancing sensory quality and sodium reduction in value-added seafood production.

## Figures and Tables

**Table 1:** Effects of thawing methods on the thawing time and moisture and salt content of reduced-salt marinated large yellow croaker.

Parameter Measurement	FM	RTT	LTT	FWT	WT
Thawing time (min)		32.57 ± 3.08 ^b^	591 ± 18.33 ^a^	14.67 ± 1.53 ^c^	
Moisture content (g/100 g)	65.87 ± 0.84 ^a^	66.45 ± 3.91 ^a^	67.21 ± 0.49 ^a^	68.61 ± 0.23 ^a^	66.73 ± 3.12 ^a^
Salt content (g/100 g)	4.21 ± 0.04 ^b^	3.95 ± 0.05 ^c^	3.85 ± 0.01 ^d^	3.49 ± 0.01 ^e^	4.30 ± 0.02 ^a^

Different superscript letters within the same row indicate significant differences among groups (*p* < 0.05). FM: fresh marinated and immediately roasted, RTT: room-temperature thawing, LTT: low-temperature thawing, FWT: flowing-water thawing, WT: frozen direct roasted.

**Table 2:** Effects of thawing methods on the texture properties of reduced-salt marinated large yellow croaker.

Texture Index	FM	RTT	LTT	FWT	WT
Hardness (N)	3.60 ± 0.15 ^cd^	3.98 ± 0.14 ^bc^	3.48 ± 0.16 ^d^	4.74 ± 0.45 ^a^	4.38 ± 0.17 ^ab^
Adhesiveness (mJ)	0.041 ± 0.006 ^d^	0.277 ± 0.029 ^a^	0.033 ± 0.008 ^d^	0.183 ± 0.012 ^b^	0.096 ± 0.010 ^c^
Cohesiveness (ratio)	0.330 ± 0.053 ^a^	0.290 ± 0.010 ^a^	0.235 ± 0.015 ^b^	0.320 ± 0.020 ^a^	0.310 ± 0.030 ^a^
Springiness (mm)	1.95 ± 0.29 ^a^	2.15 ± 0.21 ^a^	2.07 ± 0.21 ^a^	2.10 ± 0.02 ^a^	1.80 ± 0.20 ^a^
Gumminess (N)	1.24 ± 0.09 ^b^	1.47 ± 0.02 ^ab^	0.648 ± 0.092 ^c^	1.61 ± 0.29 ^a^	1.38 ± 0.19 ^ab^
Chewiness (mJ)	4.03 ± 0.25 ^a^	2.79 ± 0.47 ^c^	1.19 ± 0.09 ^d^	3.43 ± 0.39 ^b^	2.52 ± 0.11 ^c^

Different superscript letters within the same row indicate significant differences among groups (*p* < 0.05). FM: fresh marinated and immediately roasted, RTT: room-temperature thawing, LTT: low-temperature thawing, FWT: flowing-water thawing, WT: frozen direct roasted.

**Table 3:** Effects of thawing methods on the color of reduced-salt marinated large yellow croaker.

Color Parameter	FM	RTT	LTT	FWT	WT
L*	47.16 ± 1.54 ^ab^	46.99 ± 0.97 ^ab^	48.05 ± 0.21 ^a^	45.28 ± 1.63 ^b^	45.98 ± 1.78 ^ab^
a*	3.24 ± 0.04 ^c^	4.03 ± 0.54 ^bc^	3.32 ± 0.02 ^c^	5.05 ± 0.15 ^a^	4.56 ± 0.22 ^b^
b*	12.15 ± 0.47 ^a^	10.32 ± 0.71 ^abc^	11.85 ± 0.22 ^ab^	9.59 ± 1.05 ^bc^	9.20 ± 1.01 ^c^
ΔE		2.26 ± 0.27 ^b^	0.97 ± 0.16 ^c^	4.20 ± 0.27 ^a^	3.54 ± 0.68 ^a^
BI	33.93 ± 1.53 ^a^	30.36 ± 3.15 ^a^	32.51 ± 0.47 ^a^	32.08 ± 4.00 ^a^	29.17 ± 3.52 ^a^

L*: lightness/darkness, a*: redness/greenness, b*: yellowness/blueness, ΔE: total color difference, BI: browning index. Different letters within the same row indicate significant differences among groups (*p* < 0.05). FM: fresh marinated and immediately roasted, RTT: room-temperature thawing, LTT: low-temperature thawing, FWT: flowing-water thawing, WT: frozen direct roasted.

## Data Availability

The original contributions presented in the study are included in the article/Supplementary Materials. Further inquiries can be directed to the corresponding author.

## Supplementary Materials

The following supporting information can be downloaded at: https://www.mdpi.com/article/10.3390/foods14244213/s1, Table S1. Standards for sensory evaluation; Table S2. Performance description of sensors in electronic nose; Table S3. The volatile compounds and integral parameters of reduced-salt pickled large yellow croaker with different thawing treatments on GC-IMS; Table S4. Discrimination power of PCA analysis in electronic nose.
