# Supplementary material for: Effects of Thawing Methods on the Roasting Quality and Flavor Profiles of Reduced-Salt Marinated Large Yellow Croaker (Larimichthys crocea)
Source: Foods. 2025 Dec 8;14(24):4213. doi: 10.3390/foods14244213 (PMC12731519; doi:10.3390/foods14244213)
Supplement: Supplementary file 1 [file foods-14-04213-s001.zip › foods-3975474-supplementary.pdf]

Table S1. Standards for sensory evaluation.

Score	Appearance	Odor	Taste	Texture
20-25	attractive color and luster, tightly packed muscle tissue	no obvious fishy smell or off-flavor	appropriate salty and no bitter, pleasant texture	firm, good chewiness and elasticity
15-19	bright color and luster, firm muscle tissue	slightly fishy smell, but no off-flavor	appropriate salty and slight bitter, average texture	relatively firm, good chewiness and elasticity
10-14	uniform color and luster, relatively firm muscle tissue	relatively mild fishy smell and mild off-flavor	mild salty and slight bitter, uncomfortable texture	average hardness, chewiness and elasticity
5-9	relatively dull color, relatively loose muscle tissue	distinct fishy smell and slightly unusual odor	strong salty and slight bitter, unpleasant texture	relatively hard or soft, poor chewiness and elasticity
0-4	dull color, loose muscle tissue	distinct fishy and off-odor	too salty and bitter, unpleasant texture	very hard or soft, poor chewiness and elasticity

Table S2. Performance description of sensors in electronic nose.

Sensor	Detection object	Detection range（mL/m^3）
W1C	aromatic substance	methylbenzene, 10 mL/m^3
W5S	extremely sensitive to nitrogen oxides	nitrogen dioxide, 1 mL/m^3
W3C	sensitive to amines and aromatic substances	benzene, 10 mL/m^3
W6S	hydrogen sensitive	hydrogen, 100 mL/m^3
W5C	alkanes and aromatic substances	dimethyl methane, mL/m^3
W1S	methane-sensitive	methane, 100 mL/m^3
W1W	extremely sensitive to sulfur-containing compounds	sulfuretted hydrogen, 1 mL/m^3
W2S	sensitive to alcohols and aldehydes	carbon monoxide, 100 mL/m^3
W2W	sensitive to aromatic compounds and organic sulfides	sulfuretted hydrogen, 1 mL/m^3
W3S	methane-sensitive	methane, 10 mL/m^3

Table S3. The volatile compounds and integral parameters of reduced-salt pickled large yellow croaker with different thawing treatments on GC-IMS.

Compound	CAS	Molecular weight	Retention index	Retention time (s)	Drift time (ms)	FM	RTT	LTT	FWT	WT
Aldehydes										
(-)-perillaldehyde	18031-40-8	150.2	1145	629.832	1.28548	0.144 ± 0.012[a]	0.134 ± 0.024[a]	0.191 ± 0.013[b]	0.146 ± 0.005[a]	0.128 ± 0.014[a]
(E)-2-nonenal	18829-56-6	140.2	1158	660.033	1.41183	0.301 ± 0.024[ab]	0.394 ± 0.069[ab]	0.404 ± 0.036[b]	0.332 ± 0.042[ab]	0.280 ± 0.029[ab]
(E)-2-pentenal	1576-87-0	84.1	767.8	200.415	1.07521	1.737 ± 0.266[b]	0.567 ± 0.085[a]	1.738 ± 0.196[bc]	2.245 ± 0.171[c]	0.493 ± 0.084[a]
5-methylfurfural	620-02-0	110.1	950.9	324.827	1.46548	0.113 ± 0.017[a]	0.506 ± 0.046[b]	0.128 ± 0.004[a]	0.112 ± 0.015[a]	0.299 ± 0.064[b]
benzene acetaldehyde	122-78-1	120.2	1035.8	424.476	1.25892	0.125 ± 0.001[a]	0.457 ± 0.060[b]	0.122 ± 0.001[a]	0.126 ± 0.009[a]	0.561 ± 0.061[c]
butanal	123-72-8	72.1	620.3	140.462	1.27983	3.467 ± 0.404[b]	0.214 ± 0.039[a]	3.991 ± 0.718[b]	4.087 ± 0.661[b]	0.165 ± 0.030[a]
2-hexenal	505-57-7	98.1	841.2	242.575	1.51042	0.646 ± 0.086[b]	0.053 ± 0.008[a]	0.662 ± 0.057[b]	0.557 ± 0.098[b]	0.090 ± 0.023[a]
Alcohols										
(E)-2-hexen-1-ol	928-95-0	100.2	884.2	270.849	1.54949	0.348 ± 0.046[a]	0.404 ± 0.090[a]	0.437 ± 0.023[a]	0.308 ± 0.075[a]	0.223 ± 0.058[a]
(E)-3-hexen-1-ol	928-97-2	100.2	841.4	242.704	1.24799	2.667 ± 0.255[c]	0.212 ± 0.026[a]	2.414 ± 0.170[c]	2.380 ± 0.230[c]	1.130 ± 0.231[b]
β-phenethyl alcohol	60-12-8	122.2	1130.5	597.667	1.67253	0.419 ± 0.010[b]	0.207 ± 0.045[a]	0.441 ± 0.026[b]	0.398 ± 0.030[b]	0.217 ± 0.048[a]
1,3-butanediol	107-88-0	90.1	784.3	209.662	1.12708	0.637 ± 0.102[a]	0.330 ± 0.063[a]	0.453 ± 0.033[a]	0.428 ± 0.089[a]	0.397 ± 0.054[a]
1-propanol	71-23-8	60.1	566.3	125.805	1.11051	2.295 ± 0.388[b]	0.204 ± 0.014[a]	2.193 ± 0.407[b]	2.318 ± 0.477[b]	5.809 ± 0.350[c]
2-butanol	78-92-2	74.1	636.4	145.128	1.32758	13.95 ± 1.636[b]	0.228 ± 0.036[a]	12.68 ± 3.583[b]	13.02± 2.782[b]	0.244 ± 0.053[a]
2-furanmethanol	98-00-0	98.1	849.4	247.72	1.37376	0.547 ± 0.018[b]	0.116 ± 0.023[a]	0.552 ± 0.031[b]	0.553 ± 0.072[b]	0.208 ± 0.023[a]
5-methyl-2-furanmethanol	3857-25-8	112.1	930.8	307.426	1.5528	0.349 ± 0.039[a]	11.46 ± 2.158[c]	0.300 ± 0.043[a]	0.326 ± 0.037[a]	4.440 ± 0.410[b]
2-methyl-1-pentanol	105-30-6	102.2	807.7	222.628	1.29322	0.121 ± 0.011[a]	0.500 ± 0.077[ab]	0.177 ± 0.036[a]	0.129 ± 0.020[a]	1.151 ± 0.328[b]
2-propanol	67-63-0	60.1	523.1	115.219	1.22202	1.941 ± 0.244[b]	0.121 ± 0.020[a]	2.209 ± 0.405[b]	2.046 ± 0.203[b]	0.125 ± 0.004[a]

3-heptanol	589-82-2	116.2	883.6	270.457	1.32964	0.596 ± 0.069[b]	0.387 ± 0.064[a]	0.514 ± 0.020[ab]	0.535 ± 0.050[b]	0.639 ± 0.053[b]
benzyl alcohol	100-51-6	108.1	997.9	370.06	1.50108	0.139 ± 0.020[a]	0.375 ± 0.070[a]	0.130 ± 0.023[a]	0.123 ± 0.020[a]	0.170 ± 0.025[a]
methyl-3-but-3-en-1-ol	763-32-6	86.1	703.9	168.324	1.18119	2.452 ± 0.177[a]	2.591 ± 0.125[a]	2.518 ± 0.081[a]	3.527 ± 0.284[c]	3.027 ± 0.074[b]
1-pentanol	71-41-0	88.1	741.9	186.725	1.2497	0.216 ± 0.044[a]	0.063 ± 0.009[a]	0.168 ± 0.010[a]	0.210 ± 0.040[a]	0.503 ± 0.091[b]
linalool	78-70-6	154.3	1098.3	531.919	1.20805	0.231 ± 0.011[b]	0.115 ± 0.011[a]	0.241 ± 0.012[b]	0.246 ± 0.043[b]	0.132 ± 0.012[a]
Esters										
(Z)-3-hexenyl butyrate	16491-36-4	170.3	1151.4	644.542	1.41124	0.333 ± 0.042[a]	1.751 ± 0.183[b]	0.286 ± 0.002[a]	0.342 ± 0.023[a]	1.078 ± 0.130[c]
3-methylbutyl propionate	105-68-0	144.2	930.8	307.41	1.36254	0.147 ± 0.002[a]	1.078 ± 0.228[b]	0.134 ± 0.007[a]	0.158 ± 0.024[a]	1.948 ± 0.166[c]
acetic acid ethyl ester	141-78-6	88.1	586.6	131.12	1.3369	0.051 ± 0.007[a]	0.024 ± 0.002[a]	0.106 ± 0.032[b]	0.054 ± 0.005[ab]	0.019 ± 0.004[a]
butyl acetate	123-86-4	116.2	798.1	217.25	1.24045	1.451 ± 0.234[a]	1.525 ± 0.301[a]	2.208 ± 0.202[bc]	1.762 ± 0.252[ab]	2.694 ± 0.128[c]
butyl pentanoate	591-68-4	158.2	1101.4	537.973	1.39337	0.337 ± 0.048[b]	0.078 ± 0.012[a]	0.354 ± 0.056[b]	0.272 ± 0.048[b]	0.093 ± 0.019[a]
diethyl malonate	105-53-3	160.2	1080.8	499.382	1.25018	0.198 ± 0.025[b]	0.055 ± 0.010[a]	0.300 ± 0.057[b]	0.217 ± 0.046[b]	0.056 ± 0.001[a]
2-ethoxy- ethanol	110-80-5	90.1	724.7	178.16	1.09298	0.550 ± 0.039[a]	2.338 ± 0.191[c]	0.548 ± 0.032[a]	0.567 ± 0.108[a]	1.724 ± 0.311[b]
ethyl 2-methylpropa noate	97-62-1	116.2	749.7	190.764	1.20328	4.078 ± 0.328[b]	2.166 ± 0.486[a]	3.179 ± 0.176[ab]	3.621 ± 0.545[ab]	3.940 ± 0.446[b]
ethyl butyrate	105-54-0	116.2	807.6	222.59	1.19869	0.360 ± 0.004[a]	1.626 ± 0.380[b]	0.342 ± 0.028[a]	0.347 ± 0.031[a]	1.503 ± 0.067[b]
ethyl trans-2-butenoate	623-70-1	114.1	870.6	261.595	1.18312	0.259 ± 0.023[c]	0.110 ± 0.020[a]	0.220 ± 0.017[bc]	0.251 ± 0.036[c]	0.159 ± 0.016[ab]
hexyl butanoate	2639-63-6	172.3	1214.3	808.929	1.481	0.483 ± 0.058[a]	0.924 ± 0.111[b]	0.403 ± 0.020[a]	0.462 ± 0.013[a]	0.977 ± 0.101[b]
isobutyl 2-butenoate	589-66-2	142.2	986	357.784	1.32225	0.425 ± 0.034[b]	0.129 ± 0.020[a]	0.579 ± 0.053[c]	0.507 ± 0.030[bc]	0.121 ± 0.024[a]
isopentyl formate	110-45-2	116.2	767.2	200.084	1.28105	1.346 ± 0.148[a]	1.529 ± 0.356[a]	1.061 ± 0.071[a]	1.071 ± 0.131[a]	4.975 ± 0.415[b]
methyl acetate	79-20-9	74.1	564.7	125.401	1.19102	0.778 ± 0.056[ab]	0.463 ± 0.071[a]	1.497 ± 0.318[b]	0.879 ± 0.107[ab]	0.270 ± 0.056[a]
methyl octanoate	111-11-5	158.2	1132.8	602.694	1.41635	1.180 ± 0.209[b]	1.638 ± 0.432[b]	1.598 ± 0.195[b]	1.264 ± 0.306[b]	0.336 ± 0.068[a]
octanoic acid ethyl ester	106-32-1	172.3	1203.4	777.683	1.4831	0.247 ± 0.015[b]	0.091 ± 0.019[a]	0.311 ± 0.019[b]	0.259 ± 0.028[bc]	0.106 ± 0.016[a]
propionic acid hexyl ester	2445-76-3	158.2	1112.8	560.664	1.43594	1.497 ± 0.153[b]	0.201 ± 0.003[a]	1.474 ± 0.245[b]	1.211 ± 0.284[b]	0.201 ± 0.024[a]
propyl bytanoate	105-66-8	130.2	893.8	277.704	1.2681	2.149 ± 0.169[c]	1.385 ± 0.182[ab]	2.005 ± 0.152[c]	1.917 ± 0.144[bc]	0.958 ± 0.131[a]
Ketones										

2,3-butanedione	431-03-8	86.1	514.9	113.31	1.17427	2.290 ± 0.288^{b}	0.113 ± 0.024^{a}	2.499 ± 0.385^{b}	2.703 ± 0.131^{b}	0.101 ± 0.008^{a}
2,3-pentanedione	600-14-6	100.1	702.5	167.671	1.23992	0.272 ± 0.019^{a}	4.748 ± 0.798^{b}	0.223 ± 0.021^{a}	0.298 ± 0.016^{a}	1.666 ± 0.244^{a}
3-hexanone	589-38-8	100.2	798	217.191	1.48378	0.256 ± 0.016^{a}	0.363 ± 0.036^{a}	0.647 ± 0.093^{b}	0.346 ± 0.047^{a}	0.245 ± 0.007^{a}
4-nonanone	4485-09-0	142.2	1044.6	438.153	1.34576	3.195 ± 0.494^{a}	4.627 ± 0.100^{b}	3.532 ± 0.411^{a}	3.179 ± 0.435^{a}	3.244 ± 0.218^{a}
6-methylhepta-3,5-dien-2-one	1604-28-0	124.2	1125.7	587.375	1.19824	0.310 ± 0.016^{b}	0.145 ± 0.034^{a}	0.355 ± 0.029^{b}	0.339 ± 0.006^{b}	0.146 ± 0.012^{a}
acetoin	513-86-0	88.1	724.4	178.04	1.3289	5.272 ± 0.716^{b}	1.778 ± 0.301^{a}	3.595 ± 0.406^{ab}	4.369 ± 0.692^{b}	4.750 ± 0.614^{b}
acetone	67-64-1	58.1	509.5	112.075	1.11162	1.850 ± 0.295^{b}	0.130 ± 0.022^{a}	2.151 ± 0.364^{b}	2.206 ± 0.156^{b}	0.140 ± 0.005^{a}
cyclotene	80-71-7	112.1	1085	506.946	1.14569	0.743 ± 0.070^{b}	0.114 ± 0.016^{a}	0.742 ± 0.101^{b}	0.657 ± 0.084^{b}	0.249 ± 0.035^{a}
dihydro-2-methyl-3(2H)furanone	3188-00-9	100.1	797.4	216.828	1.4176	0.215 ± 0.031^{a}	2.007 ± 0.274^{c}	0.362 ± 0.053^{a}	0.307 ± 0.061^{a}	1.180 ± 0.228^{b}
dimethyldioxolone	37830-90-3	114.1	960.8	333.817	1.17428	1.292 ± 0.131^{b}	0.475 ± 0.071^{a}	1.395 ± 0.142^{b}	1.203 ± 0.293^{b}	0.280 ± 0.012^{a}
methyl isobutyl ketone	108-10-1	100.2	728.1	179.84	1.57323	0.977 ± 0.023^{b}	0.319 ± 0.019^{a}	0.937 ± 0.054^{b}	0.937 ± 0.070^{b}	0.252 ± 0.053^{a}
N-methylpyrrolidone	872-50-4	99.1	1019.8	400.549	1.43584	0.431 ± 0.043^{b}	0.163 ± 0.024^{a}	0.514 ± 0.029^{b}	0.460 ± 0.073^{b}	0.161 ± 0.036^{a}
Pyrazines										
2-acetylpyrazine	22047-25-2	122.1	1017.2	396.789	1.14139	1.816 ± 0.020^{b}	0.306 ± 0.057^{a}	1.880 ± 0.084^{b}	1.724 ± 0.323^{b}	0.536 ± 0.091^{a}
2-methyl-3-methylthiopyrazine	2882-20-4	140.2	1140.6	619.764	1.19222	0.329 ± 0.048^{a}	0.300 ± 0.065^{a}	0.311 ± 0.007^{a}	0.300 ± 0.021^{a}	0.206 ± 0.040^{a}
3-methyl-2-isobutyl pyrazine	13925-06-9	150.2	1132.4	601.819	1.81195	0.227 ± 0.033^{abc}	0.163 ± 0.012^{ab}	0.311 ± 0.044^{c}	0.243 ± 0.053^{bc}	0.134 ± 0.020^{a}
methylpyrazine	109-08-0	94.1	823.9	232.098	1.09887	0.333 ± 0.026^{a}	3.716 ± 0.225^{c}	0.567 ± 0.071^{a}	0.444 ± 0.062^{a}	1.946 ± 0.168^{b}
2-ethyl-6-methylpyrazine	13925-03-6	122.2	998.8	371.266	1.17592	0.236 ± 0.021^{a}	1.106 ± 0.269^{c}	0.178 ± 0.019^{a}	0.232 ± 0.045^{a}	0.587 ± 0.072^{b}
tetramethylpyrazine	1124-11-4	136.2	1046.6	441.278	1.68524	0.134 ± 0.018^{a}	1.256 ± 0.222^{b}	0.118 ± 0.009^{a}	0.126 ± 0.013^{a}	0.589 ± 0.108^{a}
Sulfur compounds										
4-methyl-5-vinylthiazole	1759-28-0	125.2	1044.4	437.823	1.13962	0.173 ± 0.011^{a}	1.972 ± 0.307^{b}	0.162 ± 0.015^{a}	0.166 ± 0.020^{a}	0.677 ± 0.157^{a}
4-methylthiazole	693-95-8	99.2	823.6	231.905	1.34891	0.761 ± 0.061^{b}	0.332 ± 0.034^{a}	0.807 ± 0.068^{b}	0.760 ± 0.109^{b}	0.535 ± 0.066^{ab}
allyl Isothiocyanate	57-06-7	99.2	865.8	258.401	1.09133	1.120 ± 0.213^{a}	1.014 ± 0.125^{a}	0.943 ± 0.059^{a}	1.224 ± 0.224^{a}	0.934 ± 0.205^{a}
allyl methyl sulfide	10152-76-8	88.2	692.5	163.157	1.03542	3.358 ± 0.326^{a}	4.266 ± 0.556^{a}	3.676 ± 0.380^{a}	3.327 ± 0.415^{a}	11.02 ± 0.551^{b}
dimethyl disulfide	624-92-0	94.2	763.3	197.999	1.14585	0.367 ± 0.024^{b}	0.148 ± 0.026^{a}	0.347 ± 0.040^{b}	0.359 ± 0.055^{b}	0.318 ± 0.075^{b}

1-propanethiol	107-03-9	76.2	628	142.683	1.17535	0.409 ± 0.061^{a}	3.481 ± 0.495^{c}	0.403 ± 0.050^{a}	0.476 ± 0.035^{a}	1.701 ± 0.478^{b}
Acids										
2-methylbutanoic acid	116-53-0	102.1	846	245.624	1.47563	0.518 ± 0.093^{b}	0.045 ± 0.005^{a}	0.558 ± 0.048^{b}	0489 ± 0.044^{b}	0.169 ± 0.022^{a}
2-methyl-pentanoic acid	97-61-0	116.2	930.6	307.211	1.26318	0.416 ± 0.063^{a}	3.323 ± 0.442^{b}	0.426 ± 0.063^{a}	0.384 ± 0.086^{a}	3.049 ± 0.209^{b}
3-methylbutanoic acid	503-74-2	102.1	876	265.214	1.21345	0.167 ± 0.033^{a}	0.150 ± 0.021^{a}	0.127 ± 0.017^{a}	0.172 ± 0.035^{a}	0.201 ± 0.018^{a}
acetic acid	64-19-7	60.1	594.2	133.187	1.05529	2.115 ± 0.327^{a}	3.302 ± 0.168^{a}	2.121 ± 0.233^{a}	2.092 ± 0.215^{a}	5.142 ± 1.078^{b}
allylacetic acid	591-80-0	100.1	884.9	271.341	1.4323	1.764 ± 0.034^{b}	0.673 ± 0.143^{a}	1.705 ± 0.110^{b}	1.813 ± 0.062^{b}	0.421 ± 0.096^{a}
butanoic acid	107-92-6	88.1	767.4	200.234	1.39348	1.466 ± 0.119^{a}	1.825 ± 0.325^{a}	1.873 ± 0.108^{a}	1.309 ± 0.284^{a}	3.470 ± 0.745^{b}
hexanoic acid	142-62-1	116.2	1018.7	398.904	1.6436	0.260 ± 0.030^{ab}	0.080 ± 0.004^{a}	0.385 ± 0.072^{b}	0.269 ± 0.044^{ab}	0.077 ± 0.011^{a}
pentanoic acid	109-52-4	102.1	959.8	332.913	1.5055	0.230 ± 0.042^{ab}	0.069 ± 0.010^{a}	0.317 ± 0.029^{b}	0.231 ± 0.058^{ab}	0.064 ± 0.001^{a}
Heterocycles										
2-acetyl-1-pyrroline	85213-22-5	111.1	907.6	288.458	1.47095	0.373 ± 0.033^{ab}	0.288 ± 0.056a	0.467 ± 0.053^{b}	0.431 ± 0.031^{b}	0.103 ± 0.024^{a}
2-acetyl-2-thiazoline	29926-41-8	129.2	1108.7	552.328	1.15214	1.335 ± 0.251^{bc}	0.810 ± 0.166^{ab}	1.665 ± 0.204^{c}	1.306 ± 0.263^{abc}	0.700 ± 0.067^{a}
pyrrolidine	123-75-1	71.1	716.7	174.321	1.28263	0.824 ± 0.119^{a}	2.674 ± 0.075^{c}	0.712 ± 0.021^{a}	0.759 ± 0.103^{a}	1.392 ± 0.160^{b}
Others										
1,2-dimethoxyethane	110-71-4	90.1	673.1	156.403	1.11286	1.655 ± 0.049^{a}	3.211 ± 0.410^{b}	1.291 ± 0.130^{a}	1.406 ± 0.078^{a}	2.011 ± 0.472^{a}
1-phellandrene	4221-98-1	136.2	1020	400.784	1.68246	0.490 ± 0.068^{ab}	0.102 ± 0.024^{a}	1.175 ± 0.295^{c}	0.763 ± 0.105^{bc}	0.120 ± 0.020^{a}
2-propenenitrile	107-13-1	53.1	636.9	145.293	1.09063	5.669 ± 0.851^{ab}	7.103 ± 0.896^{b}	4.159 ± 0.415^{a}	4.937 ± 0.417^{a}	5.841 ± 0.231^{ab}
dimethylacetamide	127-19-5	87.1	875.3	264.733	1.3532	0.361 ± 0.043^{a}	0.327 ± 0.070^{a}	0.319 ± 0.015^{a}	0.423 ± 0.086^{a}	0.228 ± 0.036^{a}
ethylbenzene	100-41-4	106.2	827.9	234.444	1.07133	1.298 ± 0.205^{a}	1.392 ± 0.096^{a}	1.263 ± 0.067^{a}	1.312 ± 0.093^{a}	2.119 ± 0.116^{b}
n-butylcyclohexane	1678-93-9	140.3	1008.2	384.101	1.25648	0.408 ± 0.022^{b}	0.139 ± 0.026^{a}	0.463 ± 0.053^{b}	0.472 ± 0.030^{b}	0.107 ± 0.024^{a}
tetrahydrofuran	109-99-9	72.1	629.3	143.051	1.22222	1.108 ± 0.117^{ab}	2.457 ± 0.334^{b}	1.557 ± 0.277^{ab}	1.528 ± 0.329^{ab}	0.516 ± 0.121^{a}
triethylenediamine	280-57-9	112.2	1019.5	400.079	1.51058	0.282 ± 0.037^{b}	0.098 ± 0.006^{a}	0.295 ± 0.047^{b}	0.298 ± 0.032^{b}	0.094 ± 0.006^{a}
undecane	1120-21-4	156.3	1125.4	586.784	1.10014	3.585 ± 0.306^{cd}	2.133 ± 0.185^{b}	3.195 ± 0.125^{c}	3.906 ± 0.261^{d}	0.988 ± 0.080^{a}

Table S4. Discrimination power of PCA analysis in electronic nose.

Sample	FM	RTT	LTT	FWT
FM		0.565	0.326	0.369
RTT	0.565		0.315	0.639
LTT	0.326	0.315		0.348
FWT	0.369	0.639	0.348	
WT	0640	0.671	0.647	0.581
